# Supplementary material for: Determinants of obstructed labour and its adverse outcomes among women who gave birth in Hawassa University referral Hospital: A case-control study
Source: PLoS One. 2022 Jun 24;17(6):e0268938. doi: 10.1371/journal.pone.0268938 (PMC9231795; doi:10.1371/journal.pone.0268938)
Supplement: S1 File — (DOCX) [file pone.0268938.s001.docx]

Supplementary file 1: Sample size determination of outcomes of obstructed labour, 2018

| Outcomes of obstructed labour | Maternal complication | Perinatal mortality |
| --- | --- | --- |
| Hypothetical Proportion of outcome in nonobstructed | 1.4 % | 6.5% |
| Hypothetical Proportion of outcome in obstructed | 10.2% | 16.34 |
| Least extreme odds ratio | 8 | 2.61 |
| Cases | 90 | 155 |
| Controls | 179 | 309 |
| Total Sample size | 269 | 486 |
