# Supplementary material for: Determinants of obstructed labour and its adverse outcomes among women who gave birth in Hawassa University referral Hospital: A case-control study
Source: PLoS One. 2022 Jun 24;17(6):e0268938. doi: 10.1371/journal.pone.0268938 (PMC9231795; doi:10.1371/journal.pone.0268938)
Supplement: Attachment — Submitted filename: Reviewer respnose for Plos .docx [file pone.0268938.s004.docx]

**Dear editors and reviewers**

We would like to extend our deepest appreciation for devoting your time to review our manuscript entitled " Determinants of obstructed labor and its adverse outcomes among women who gave birth in Hawassa University Referral Hospital: A case control study”. Globally, obstructed labour accounted for 12% of maternal death and most of the maternal morbidity and perinatal mortality. It is one of the most common preventable causes of maternal and perinatal morbidity and mortality in developing countries.

Many of deaths and morbidities due to obstructed labour are entirely preventable and treatable. For this, the Ethiopian government had performed different activities. However, studies showed that adverse feto- maternal outcomes appear to be high and a common challenge in Ethiopia , the leading cause of maternal mortality in the with uterine rupture. Even though, the predictors associated with obstructed labour are scarce in Ethiopia. Determining the determinants of obstructed labor, maternal and perinatal outcomes of obstructed labour in our setting remains paramount to reduce the mortality and its morbidity. This study may be used for policy makers and a means of achieving the SDG target fetomaternal deaths from 2030. Therefore, this study was aimed to assess determinants of obstructed labor and its adverse outcomes of obstructed labor among women who gave birth in Hawassa teaching hospital, Southern Ethiopia.

Dear reviewer, there has been a major revision of this manuscript (Abstract, introduction, methods, results discussion and conclusions). The language has been extensively examined to correct grammatical and spelling inconsistencies and the whole structure of the manuscript has been revised. We hope now the manuscript is clear and more acceptable than its previous version. We have tried to present the paper in proper manner according to your comment what to supposed to do so. For this, here we have given our responses to each of the concerns you raised, highlighted by red color. Again, we would like to remind our strongest gratitude for your effort for the improvement of this manuscript and all the points were addressed below.

**Regards**

Reviewer # 1

General comment: authors should try and correct the spelling and grammatical errors.

Response: thank you for your scholarly comments and suggestions.

Abstract: In the methods area mention should be made of the fact that the study was based on patient records/charts.

Response: accepted, the fact that the study was based on patient records/charts was putted.

Background: authors ought to refer to a similar study conducted by Mulugeta et al. 2020. Reasons as to why the study was done in Hawassa?

Response: Thanks for the scholarly comments. Actually, the paper published by Mulugeta D et al. was published in 2020, access in the year 2020, while our study was conducted in 2018. Again, there was difference in the study setting in which that was mulugeta’s was in Amhara region referral hospitals and ours was in Southern region.

Discussion: Please authors include reference to a study by Mulugeta Dile et al. (2020) titled: Determinants of obstructed labour among women attending intrapartum care in Amhara Region NW Ethiopia: a hospital based unmatched case-control study.

Response: accepted and revision was made.

In the limitations there is no mention about missing data which is common in use of secondary data.

Response: accepted and it is already included as “The retrospective nature of the study might prevent data collection for some variables like educational level, type of delays, socioeconomic status, nutritional status, and infrastructure of the health facilities as these variables were not registered in women’s obstetric cards.”

Conclusion: Strong recommendation on the use of family planning though relevant but does not come directly from the data.

Response: Accepted, but the recommendations is an area of implications based on the findings. Hence, low birth order is a prevention for obstructed labor, top reduce multiparity utilization of family planning is paramount and better to be recommended.

Reviewer #2: 1. Generally, the article is very important to share with the wider community as it addresses determinants of obstructed labour which is one of the direct causes of maternal deaths. Knowing the determinants of obstructed labour will help to put measures that will improve maternal health and ultimately reduce maternal mortality.

Response: thank you for your interest on the area of interest.

2. The abstract is more than 300 words based on journal guidelines, try to reduce redundant words.

Response: accepted and revision was made.

3. There were a lot of grammatical errors throughout the documents. Needs to revise the whole document.

Response: Thank you very much and overall revision of the document was made.

4. Background: The authors need to unpack and describe all the determinants of obstructed labour reported previously in other studies in the background section.

Response: accepted and remained determinants were incorporated.

5. Ethic: In Ethics statement line 4, correct repeated word, “consent was”

Response: accepted and repeated word was removed.

6. Sample size: The sample size calculation is not well computed; This is a case control study and decided to take a ratio of 1:2, how did you arrive with a total number of 486 women (162 women for cases and 324 women for controls)? This should be well elaborated.

Response: description of sample size calculation was narrated.

7. Methods: In the Method section, under “Study design, setting and population” line 14...This sentence is not clear, “All women who gave birth after 28 weeks of gestation or weight of at least 1000 gm were included in the study”.

Response: we tried to explain to describe the deliveries after viability in Ethiopia context that is deliveries after 28 weeks or live births weights 1000 gm.

8. Discussion: In this section: For the reader to follow, it is best to give the summary of key important findings in the first paragraph, and in the subsequent paragraphs to continue discussing your findings by comparing with other previously reported studies. It is also important to minimize repeating describing your findings in the discussion section. The discussion of your findings should be explicitly discussed since then all your results have been well elaborated in your results section. Eg. “The absence of partograph utilization observed in this study as one of the major determinant factors of OL was also reported in other studies in Ethiopia”. In addition, avoid reporting the odds ratio, percentages ( %), CI, P-Values in the discussion part. Just report in wording the interpretation of your findings and compare them with other previous studies. Prefer to speak, half of.., a third of.. Rather than 50.3%, etc.

Response: Thank you for the highly scholarly comments and revision was made. We have putting the interpretation of the findings and compare them with other previous studies and the odds ratio, percentages ( %), CI, P-Values in the discussion part is avoided.

9. Some abbreviations have not been fully expressed at first use like “NICU”

Response: accepted and revision was made.

10. For Table 3: (Determinants of obstructed labour in Hawassa Referral Hospital, Ethiopia); since it is long, no need for the subheading, just leave it for it to continue as it is.

Response: accepted and subheadings were removed from the Table 3.

12. Maintain continuity of the layout of all tables as instructed by the journal, Table 4 has a different layout compared with other tables.

Response: accepted and revision was made. we have make the layout of Table 4 similar with other tables seems like.

13. It is advisable to follow the journal guidelines: For the PLOS one, the manuscript should be double spaced, with line numbers etc…

Response: accepted and revision was made and line number was provided.

14. Revise your reference list to be consistent, eg. Number 50. The authors' list has small capitals.
