# Supplementary material for: Determinants of obstructed labour and its adverse outcomes among women who gave birth in Hawassa University referral Hospital: A case-control study
Source: PLoS One. 2022 Jun 24;17(6):e0268938. doi: 10.1371/journal.pone.0268938 (PMC9231795; doi:10.1371/journal.pone.0268938)
Supplement: Attachment — Submitted filename: Determinants of obstructed labour_comments.doc [file pone.0268938.s005.doc]

**Determinants of obstructed labour and its adverse outcomes among women who gave birth in Hawassa University Referral Hospital: A case-control study**

**Minor comments**

1. Congrats to the authors for improving the manuscript. However, some of the comments from the reviews have not been satisfied.

2. The abstract is still more than 300 words. The abstract has a number of irrelevant words which could be omitted.Eg, in Methods paragraph, the words like… “ odds ratio/CI/ Statistical significance was declared when the p-value was less than 0.05” are not necessary to mention here. I will advise the authors to revisit the abstract again to be clear, short and precise to comply with the journal requiremnts.

3. Some grammatical errors and tenses have not yet been satisfied, eg. “…sample size is …” instead of “…sample size was….”. Revise the whole document to clear all the minor spelling and tenses.
