# Supplementary material for: Determinants of obstructed labour and its adverse outcomes among women who gave birth in Hawassa University referral Hospital: A case-control study
Source: PLoS One. 2022 Jun 24;17(6):e0268938. doi: 10.1371/journal.pone.0268938 (PMC9231795; doi:10.1371/journal.pone.0268938)
Supplement: Attachment — Submitted filename: Final response for Plos .docx [file pone.0268938.s006.docx]

**Dear editors and reviewers**

We would like to extend our deepest appreciation for devoting your time to review our manuscript entitled " Determinants of obstructed labor and its adverse outcomes among women who gave birth in Hawassa University Referral Hospital: A case control study”. Globally, obstructed labour accounted for 12% of maternal death and most of the maternal morbidity and perinatal mortality. It is one of the most common preventable causes of maternal and perinatal morbidity and mortality in developing countries.

Many of deaths and morbidities due to obstructed labour are entirely preventable and treatable. For this, the Ethiopian government had performed different activities. However, studies showed that adverse feto- maternal outcomes appear to be high and a common challenge in Ethiopia , the leading cause of maternal mortality in the with uterine rupture. Even though, the predictors associated with obstructed labour are scarce in Ethiopia. Determining the determinants of obstructed labor, maternal and perinatal outcomes of obstructed labour in our setting remains paramount to reduce the mortality and its morbidity. This study may be used for policy makers and a means of achieving the SDG target fetomaternal deaths from 2030. Therefore, this study was aimed to assess determinants of obstructed labor and its adverse outcomes of obstructed labor among women who gave birth in Hawassa teaching hospital, Southern Ethiopia.

Dear reviewer, there has been a minor revision of this manuscript. The reference list has been extensively reviewed to ensure its completeness and to correct inconsistencies. We hope now the manuscript is clear and more acceptable than its previous version. We have tried to present the paper in proper manner according to your comment what to supposed to do so. For this, here we have given our responses to each of the concerns you raised, highlighted by red color. Again, we would like to remind our strongest gratitude for your effort for the improvement of this manuscript and all the points were addressed below.

**Regards**

1. Congrats to the authors for improving the manuscript. However, some of the comments from the reviews have not been satisfied.

Response: Thank you for your comments, even we tried to further reviewed it again.
2. The abstract is still more than 300 words. The abstract has a number of irrelevant words which could be omitted. Eg, in the Methods paragraph, words like… “Odds ratio/CI/ Statistical significance was declared when the p-value was less than 0.05” are not necessary to mention here. I will advise the authors to revisit the abstract again to be clear, short, and precise to comply with the journal requirements.

Response: Thank you for your scholarly comments and abstract was revised.
3. Some grammatical errors and tenses have not yet been satisfied, eg. “…sample size is …” instead of “…sample size was….”. Revise the whole document to clear all the minor spelling and tenses.

Response: Thank you for the comments and revised was made based on the comment.

**Regards**
